# Long-Term Course of Polymorphic Light Eruption: A Registry Analysis

**DOI:** 10.3389/fmed.2021.694281

**Published:** 2021-07-16

**Authors:** Alexandra Gruber-Wackernagel, Tanja Schug, Thomas Graier, Franz J. Legat, Hanna Rinner, Angelika Hofer, Franz Quehenberger, Peter Wolf

**Affiliations:** ^1^Research Unit for Photodermatology, Department of Dermatology, Medical University of Graz, Graz, Austria; ^2^Institute for Medical Informatics, Statistics and Documentation, Medical University of Graz, Graz, Austria

**Keywords:** polymorphic light eruption, disease course, persistence, predictive factors, remission

## Abstract

**Background:** Little is known about the long-term course of polymorphic light eruption (PLE).

**Objective:** To predict disease course, a questionnaire was sent to patients whose PLE had been diagnosed between March 1990 and December 2018 and documented in the Austrian Cooperative Registry for Photodermatoses.

**Methods:** In January 2019, 205 PLE patients were contacted by mail and asked to complete a questionnaire on their disease course, including whether the skin's sun sensitivity had normalized (i.e., PLE symptoms had disappeared), improved, stayed the same, or worsened over time. Patients who reported normalization of sun sensitivity were asked to report when it had occurred.

**Results:** Ninety-seven patients (79 females, 18 males) returned a completed questionnaire. The mean (range) duration of follow-up from PLE onset was 29.6 (17–54) years for females and 29.4 (16–47) years for males. The disease disappeared in 32 (41%) females after 17.4 (2–41) years and in 4 (24%) males after 11.8 (5–26) years. Twenty-nine (37%) females and 6 (35%) males reported improvement of symptoms over time; 15 females (19%) and 7 males (41%) reported no change; and 3 females (4%) and no males reported worsening of symptoms. Kaplan-Meier analysis revealed that after 20 years 74% (95%CI, 64–82%) of patients still suffered from PLE. PLE lesion persistence (>1 week) tended to predict a prolonged course of PLE.

**Conclusions:** PLE usually takes a long-term course over many years though in most patients its symptoms improve or disappear over time. How improvement relates to the pathophysiology of the disease remains to be determined.

## What is Already Known About this Topic?

Polymorphic light eruption (PLE) has a long-term course.

## What Does this Study Add?

PLE symptoms improve or disappear over time in approximately three quarters of patients although it takes 20 years until one quarter of patients has normalized from the disease. A long persistence of PLE lesions under daily life conditions may predict a poor prognosis for clinical disease remission.

## Introduction

Polymorphic light eruption (PLE) is the most common and prevalent photodermatosis, particularly among young women in temperate climates (1–4). In a pan-European study, the average PLE prevalence was 18% (4). Similar to autoimmune diseases, PLE affects women approximately four times more often than men and usually has its onset within the first three decades of life (2, 5, 6). Several hours to days after initial exposure to intense sunlight, usually in spring or early summer, itchy skin lesions of variable morphology appear on sun-exposed skin. Many patients also experience flares during summer holidays (4). If further sun exposure is avoided, skin lesions subside without scarring within days. However, repeated exposure to sunlight reduces susceptibility to PLE. As summer progresses, many individuals experience a hardening effect after repeated exposure (2, 7, 8), making skin lesions less likely to occur or less severe. Unfortunately, this natural photohardening effect as well as the hardening effect of prophylactic medical phototherapy are lost in winter; consequently, PLE lesions recur the next year and often for years to come (2, 9, 10).

Recently, the pathophysiology of PLE has become much better understood. This includes initial triggers (11–14); concurrent resistance against induction of UV-induced immune suppression, linked to an imbalanced micromilieu marked by low levels of IL-4, IL-10, and TNF-alpha (15, 16); failure of Langerhans cell emigration from the skin and neutrophilic infiltration into the skin (17–21); disturbances in Treg levels and function (22–24); and potential involvement of CD11b/IL-31+ cells (25), mast cells (26, 27) or plasmacytoid dendritic cells (28, 29). Also better understood now are the therapeutic mechanisms of photohardening (19, 23, 27, 30–34) and other preventive measures (35–38). However, little is known about the initial and long-term course of the disease.

The aim of our study was to investigate the course of PLE and to identify potential predictive factors for the course and duration of the disease. Data for the analysis were available from standard questionnaires collected over a period of 30 years on a routine basis from patients with PLE and documented in the Cooperative Registry for Photodermatoses at the Medical University of Graz. In order to identify potential predictive factors for the course of the disease, patients were invited to report in an additional new questionnaire the course of their disease over the years and whether symptoms had improved, vanished or worsened.

## Patients and Methods

### Study Setting

This study and the Austrian Cooperative Registry for Photodermatoses from which its data were extracted were approved by the ethics committee of the Medical University of Graz (application no. 30-089 ex 17/18). All patient data recorded in the registry were extracted from patient charts (paper or electronic) and from parts of a questionnaire designed for patients with photodermatoses that was routinely completed by those visiting the Outpatient Photodermatology Unit, Medical University of Graz. The questionnaire contained questions concerning patient demographics and disease characteristics. A key question for this study was whether the skin's sun sensitivity disappeared, improved, stayed the same, or worsened over time. If the answer was normalization of sun sensitivity (i.e., cessation of PLE symptoms), the patient was asked to report when or over what time interval the normalization had occurred.

### Study Population

**Figure 1:**
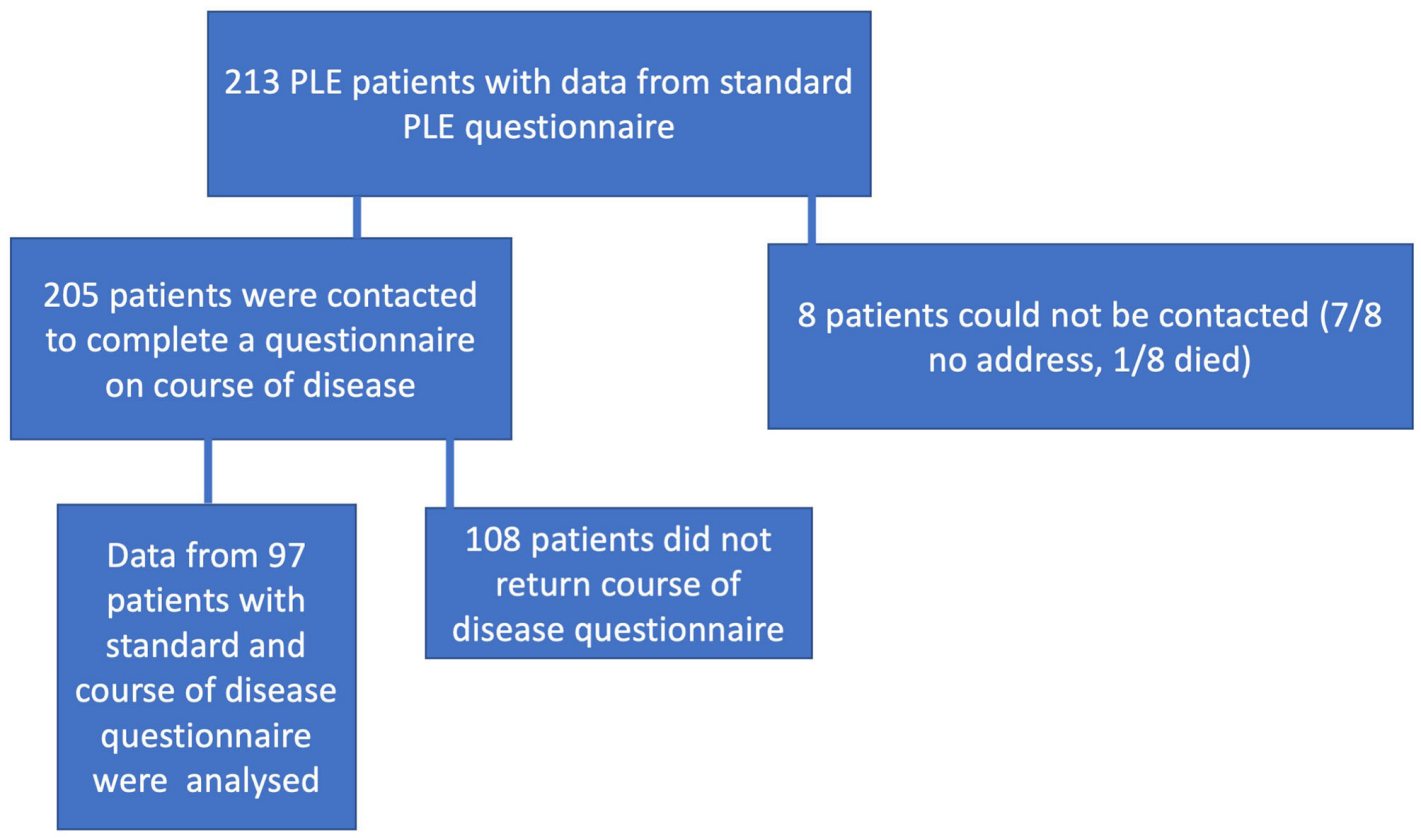
Patient flow chart.

In January 2019, 205 of 213 patients who were already enrolled in the Cooperative Registry for Photodermatoses and who had visited our Outpatient Photodermatology Unit between March 1990 and December 2018 were contacted by mail and asked to complete a questionnaire on their disease course. Ninety-seven of them (79 females and 18 males) returned completed questionnaires and their data were analyzed. The flow chart showing patient selection is presented in Figure 1.

### Statistical Analysis

Descriptive statistics were used to report and compare patient demographics. An unpaired, two-tailed Student's *t*-test, chi-square test, or Fisher exact test was administered to determine statistical differences between females and males with PLE. Persistence of PLE was analyzed by survival analysis, considering normalization of PLE as event of interest. Patients who had not normalized until the completion of the questionnaire were considered as censored. In order to assess risk factors for normalization Kaplan-Meier curves and univariate and multivariate Cox models were calculated. The logrank test criterion was used. The patient or disease characteristics under study were sex, age at disease onset, skin phototype, seasonal occurrence of skin lesions, and lesion occurrence (within 24 h after sun exposure) and duration (more than 1 week). Statistical analyses were performed and graphical illustrations were created using Prism 6 for Mac OSX V 6.0f, USA, GraphPad Prism V.8.4.1, USA and SPSS V25.0.0.1, IBM, USA, and R 4.0.2 (www.r-project.org) using packages survival 32-11 and survminer 0.4.9.

## Results

### Patient Characteristics

**Figure 2:**
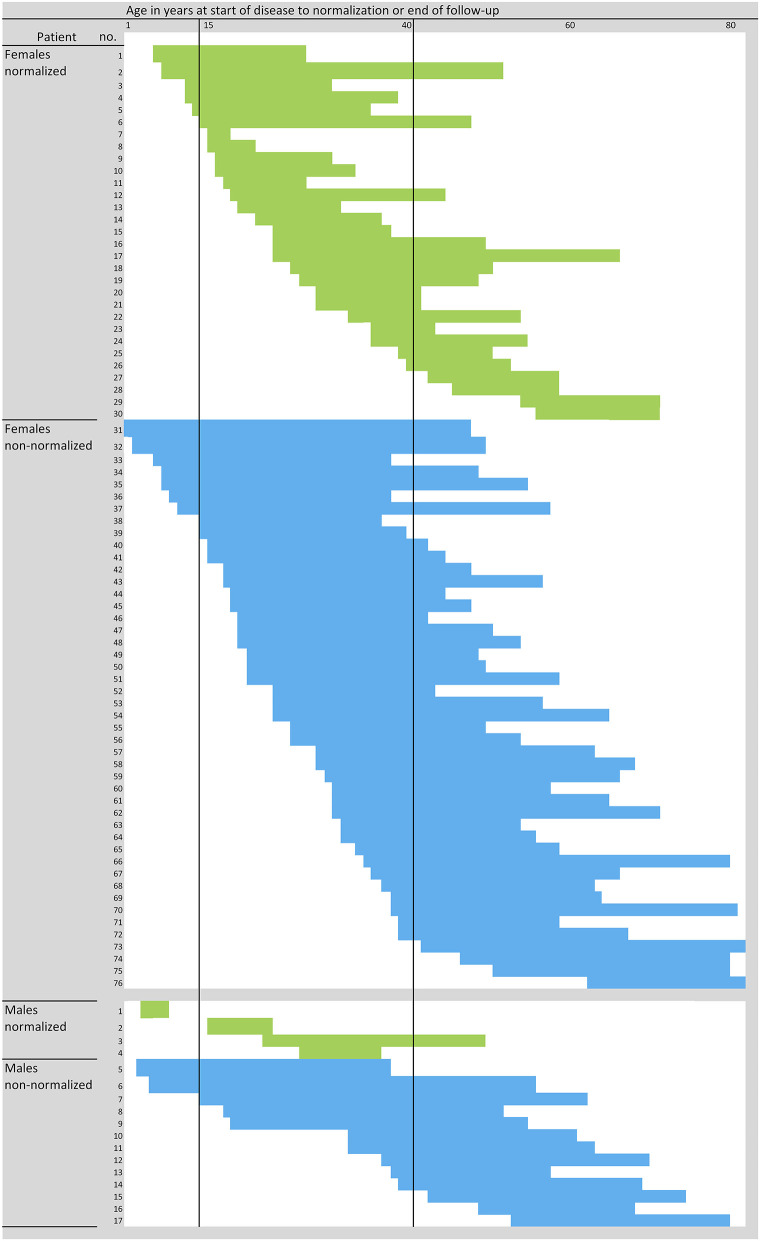
Course of disease in individual PLE patients. Blue and green represent individual patients and their time span from onset of disease to normalization of sun sensitivity (i.e., cessation of PLE symptoms) (green), being considered as event, or the end of the follow-up (not-normalized, blue), being considered as censored. *N* = 97 patients; three women did not exactly report the start and/or cessation or improvement of symptoms, and one man did not answer the question on the course of the disease at all, and thus the data for these four patients were not plotted.

Our study population for data analysis included 97 patients, most of them (81.4%) female (Table 1). Except for body site distribution of PLE lesions, there were no statistically significant sex-specific differences in demographics, disease characteristics, or follow-up. The mean age at disease onset was 25.9 years for females and 28.1 years for males (Table 1). Disease onset occurred between age 15 and 40 years in most females (56/76, 74%) and most males (11/17, 65%; Figure 2). However, it occurred before age 15 years in 12 (16%) females and 3 (18%) males. This included a boy who was 5 years old at disease onset and 8 years old at initial (and effective) prophylactic photohardening with 311-nm narrowband UVB light. Disease onset occurred after age 40 years in 8 (11%) females and 3 (18%) males (Figure 2). By sex, the most common morphological type of PLE was macular in females (63%) and papular in males (60%), with overlap among the other different morphological types in many patients (Table 1). Significantly more females than males reported PLE involvement of the V-neck (90 vs. 39%; *p* < 0.0001) (Table 2). Females tended to have a lower skin phototype than did men (type I/II, 44 vs. 7%; *p* = 0.0671) (Table 1). Results of antinuclear antibody (ANA) serum testing were available for 37 females and 8 males. Apart from one female (ANA titer of 1:80) and one male (ANA titer of > 1:80), all patients had negative test results. The clinical follow-up (including testing for antinuclear antibodies) revealed no suspicion for LE in any patients with a long persistence of skin lesions included in this study (data not shown).

**Table 1:** Patient characteristics.

	**Females**	**Males**	***p*****-value**
Number/total number of patients (%)	79/97 (81.4%)	18/97 (18.6%)	
Age at disease onset (years), median, mean (SD), range	24.0 25.9 (±12.4) 1–62	30.0 28.1 (±15.0) 4–53	0.534
Age in years at providing the standard questionnaire: median, mean (SD), range	34.0 35.01 (±11.6) 15–64	37.5 35.6 (±11.2) 9–55	0.846
Skin phototype, number (percentage)	I 4 (5%) II 31 (39%) III 39 (49%) IV 5 (6%) Na 0	I 0 (0%) II 1 (7%) III 11 (79%) IV 2 (14%) Na 4	0.067
Type of PLE, number (percentage) patients	Mac: 48 (63%) Ves: 23 (30%) Pap: 29 (38%) Urt/plaq: 39 (51%) Na 3	Mac: 8 (53%) Ves: 4 (27%) Pap: 9 (60%) Urt/plaq: 7 (47%) Na 3	0.636
Lesions occurring in spring, summer, fall, winter. Number (percentage) patients	Spring 35 (49%) Summer 67 (94%) Fall 11 (15%) Winter 7 (10%) Na 8	Spring 6 (50%) Summer 12 (100%) Fall 1 (8%) Winter 3 (25%) Na 6	0.551
Persistence of skin lesions, hours (≤24 h), days (>1d-≤7d), weeks (>7d)	Hours: 19 (26%) Days: 43 (58%) Weeks: 12 (16%) Na 5	Hours: 1 (8%) Days: 7 (54%) Weeks: 5 (38%) Na 5	0.113
Occurrence of skin lesions within hours (≤24 h), days (>24 h)	Hours: 47 (69%) Days: 21 (31%) Na 11	Hours: 10 (91%) Days: 1 (9%) Na 7	0.169

*Mac, macular; Ves, vesicular; Pap, papular; Urt/plaq urticarial/plaques.; Na, no answer. P-values were determined by student's t-test and chi-square, or Fisher's exact test, whatever was the most appropriate*.

**Table 2:** Body site involvement in PLE.

**Body site of PLE involvement**	**Females (*n* = 79)**	**Males (*n* = 18)**	***p*****-value**
Face	25 (32%)	4 (22%)	0.5714
V-neck	70 (90%)	7 (39%)	**<0.0001**
Neck	24 (31%)	6 (33%)	0.9999
Back	19 (24%)	8 (44%)	0.1431
Upper chest	32 (41%)	11 (61%)	0.1878
Abdomen	21 (27%)	5 (28%)	0.9999
Upper arm	42 (54%)	7 (39%)	0.3017
Forearm	46 (59%)	13 (72%)	0.4217
Thigh	29 (37%)	6 (33%)	0.9999
Lower leg	32 (41%)	7 (39%)	0.9999
Dorsum hand	24 (31%)	6 (33%)	0.9999
Dorsum feet	22 (28%)	5 (28%)	0.9999
na	1	0	na

*na, not available. P-values were determined by chi-square, or Fisher's exact test, whatever appropriate. Significant rates are printed in bold*.

### Disease Course and Prognostic Factors

Data on disease course are presented in Table 3. The mean (range) follow-up period (from disease onset to last follow-up) was 29.6 (17–54) years for females and 29.4 (16–47) years for males. Thirty-two females (41%) and four males (24%) reported normalization of sun sensitivity (i.e., cessation of PLE symptoms) after a mean time of 17.4 (2–41) years and 11.8 (5–26) years, respectively. In those patients, the mean disease-free observation period was 12.2 (2–24) years for the females and 15 (10–21) years for the males (Table 3).

**Table 3:** Course of PLE.

	**Females**			**Males**		
**Course of disease**	**Number (%)**	**Years until cessation of disease: median, mean, range**	**Years of follow up: median, mean, range**	**Number (%)**	**Years until cessation of disease: median, mean, range**	**Years of follow up, median, mean, range**
Worse symptoms	3 (4)		30.0 31.0 23–40	0 (0)		
Equal symptoms	15 (19)		32.0 31.3 17–43	7 (41)		29.0 33.3 24–47
Less symptoms	29 (37)		26.5 28.7 18–45	6 (35)		30.0 26.5 17–32
Normalized	32 (41)	15.5 17.4 2–41	28.0 [11.5]^*^ 19.5 [12.2] 17–54 [2–24]	4 (24)	8.0 11.8 5–26	23.5 [14.5] 26.8 [15.0] 16–44 [10–21]
All courses	79 (100)		28.0 29.6 17–54	17 (100)		29.0 29.4 16–47
na	0			1		

* *Numbers in square brackets indicate time of follow up after cessation of disease. na, no answer available. One man did not answer the question on the course of the disease, and two women did not report the start; one woman did not report the time of cessation of symptoms, and thus the data for these four patients could not be included in the analysis of the follow-up and disease duration (see also footnote in Figure 2)*.

**Figure 3:**
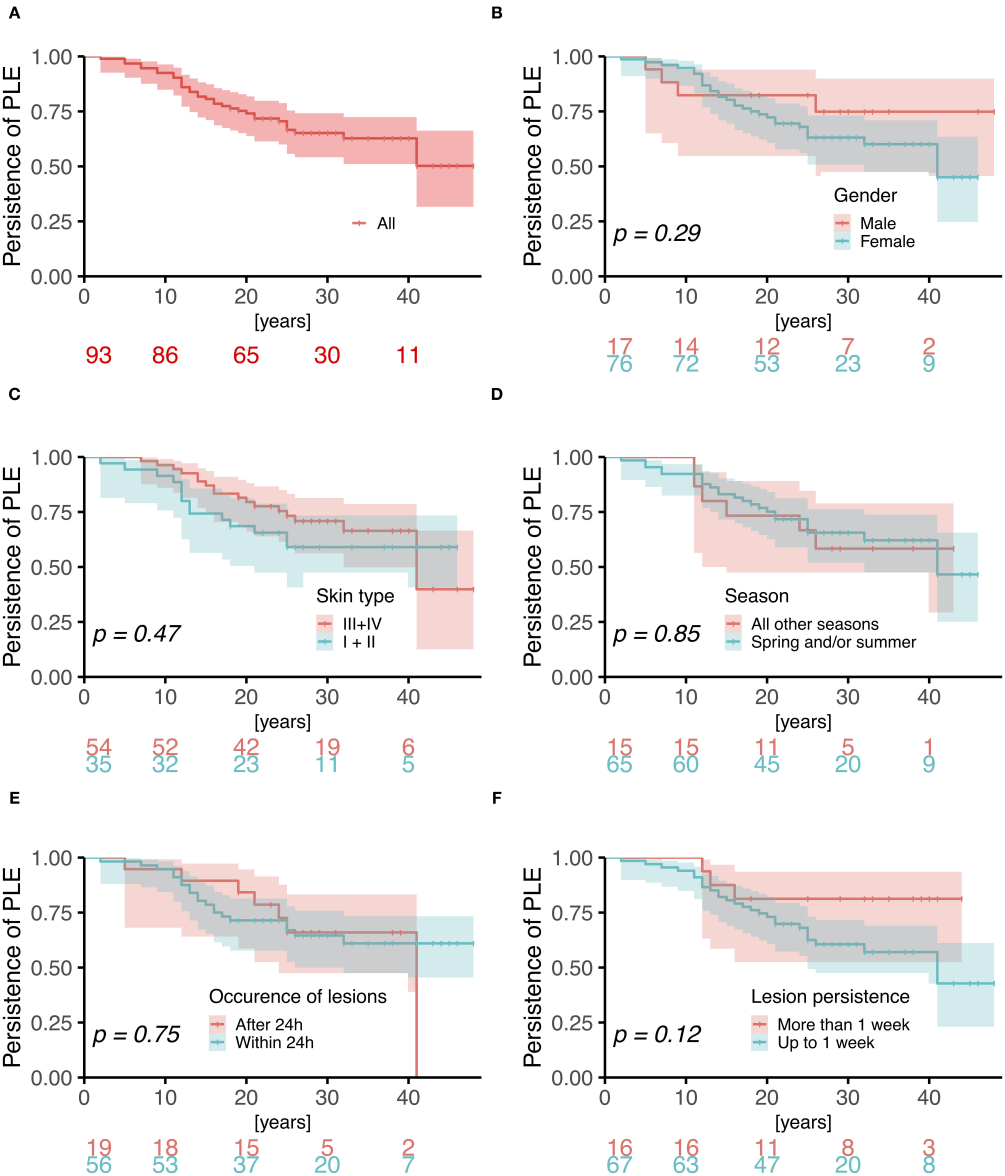
Kaplan-Meier analysis of persistence of PLE. Log-rank *p*-values are blotted in the individual graphs for **(A)** all patients and comparing **(B)** male vs. female gender (hazard ratio 1.75; 95%CI, 0.62–4.97), **(C)** skin phototype III/IV vs. I/II (1.29; 0.64–2.61), **(D)** PLE lesions during all seasons vs. spring/and or summer (0.92; 0.38–2.26), **(E)** occurrence of lesions after 24 h vs. within 24 h (0.86; 0.38–1.96), and **(F)** lesion persistence of more than 1 week vs. up to 1 week (2.47; 0.75–8.13).

Twenty-nine (37%) females and 6 (35%) males reported improvement of symptoms over time; 15 females (19%) and 7 males (41%) reported no change; and 3 females (4%) and no males reported worsening of symptoms. The long-term duration of PLE symptoms is plotted for individual patients in Figure 2. Persistence of PLE was analyzed by survival analysis and results are blotted in Figure 3. After 20 years 74% (95%CI, 64–82%) of patients still suffered from PLE. No median time of persistence could be given as the lowest point of the Kaplan-Meier curve was at 52%. However, it took 20 (95%CI, 13–26) years until one quarter of patients had normalized from PLE. It took 25 (95%CI, 18–41) years until one third of patients had normalized from PLE. Univariate and multivariate analysis (after Bonferroni *p*-value adjustment) revealed no statistical significance for the patient characteristics under study including sex, age at disease onset, skin phototype, seasonal occurrence of skin lesions, and lesion occurrence after sun exposure (Figure 3; data for age at disease onset not shown). However, there was a trend for PLE lesion persistence (more than 1 week) predicting a prolonged course of PLE (Figure 3F). The hazard ratio for lesion persistence was 2.47 (95%CI, 0.75–8.13). There was no statistical significance in the omnibus test for the set of risk factors in consideration (*p* = 0.3).

## Discussion

Previous studies have shown that in most PLE-affected subjects the disease is persistent and slow to improve (39, 40). Jansen et al. contacted patients of a cohort 7 years after an original study and reported a significant reduction of sun sensitivity in 64 of 114 subjects (56%), including 12 subjects (11%) who achieved total absence of appearance of lesions over time (40). In a subsequent study of the same cohort with a mean follow-up duration of 32 years after the onset of PLE, 23 of 94 (24%) became disease free, 48 (51%) experienced improvement of symptoms (less frequent or severe), and 23 (24%) showed equal or worse symptoms (39).

In our study, PLE symptoms vanished in 32 of 79 females (41%) and 4 of 17 males (24%) after a mean disease duration of 17 and 12 years, respectively (Table 3). When improvement in PLE symptoms was included, those rates increased to 61 of 79 females (77%) and 10 of 17 males (59%) (Table 3). However, Kaplan-Meier analysis revealed that in overall it took 20 years until one quarter of patients had normalized from PLE and 25 years until one third of patients had normalized from PLE. There was trend for PLE lesion persistence (>1 week) predicting a prolonged course of PLE by a hazard ratio of 2.47 (95%CI, 0.75–8.13) (Figure 3F). How this relates to the pathophysiology of PLE such as disturbed neutrophil infiltration (41) remains to be determined. Meanwhile, a longer PLE lesion persistence may indicate a pathophysiologic relationship with lupus erythematosus (LE). Indeed, some groups have suggested that PLE and LE share a common pathogenesis and that PLE can progress to LE (42–44). However, long term follow up studies of PLE patients have shown no increased risk of transition to LE (39, 40), although PLE lesions may precede the development of LE (45). Photosensitivity is one of the pathognomonic features of LE, and in some cases the sun-related skin rash seen in lupus can be virtually indistinguishable from PLE (42, 45–47). However, in our study, results of ANA testing were negative in all patients (except for one patient of each sex), and follow-up revealed no instances of suspected LE in any patients, including those with persistent skin lesions.

Our study also indicated that disease onset usually occurred between young adulthood and middle age, at a mean age of 25.9 years in females and 28.1 years in males (Table 1). Moreover, we found that in most cases (74% of females and 65% of males), the onset of disease (PLE symptoms) occurred between the ages of 15 and 40 years (Figure 2). Whether the quality or quantity of the microbiota present on humans during different periods of life plays a role in this needs to be determined. We recently hypothesized a potential link between disturbances in the microbiome and UV-induced immune suppression (48–51) and PLE formation (11) and reported an age-dependent skin microbiota and a potential role of sex hormones (52) and cited herein.

Consistent with previous studies, the female/male ratio in our study (4.39) was high (2, 10, 39). However, there were no significant sex-based differences other than in the body site of PLE involvement. Significantly more females than males reported V-neck lesions (90 vs. 39%; *p* ≤ 0.0001) (Table 2). This difference may be due to sex-based differences in seasonal changes in clothing style and exposure of the skin of this body site. Perhaps women experience more sudden changes in clothing style and exposure in spring, after a long fall and winter, resulting in more frequent occurrence of V-neck lesions. Alternatively, a difference in how women and men perceive this body site may account for the reported difference.

Fitzpatrick skin phototype has been associated with the likelihood of developing PLE, skin type I posing the highest risk and skin type IV or higher posing the lowest (4). In our study, most patients had skin type III (49% of females and 79% of males), and skin type I/II was not a significant predictor of PLE course (Figure 3). Moreover, age at onset of disease, sex, and occurrence of lesions in certain seasons (spring and/or summer exclusively) and the time period of occurrence of lesions after sunlight exposure also had no predictive value with regard to the course of the disease (Figure 3).

Our study had several limitations. One was the relatively low rate of return of completed disease-course questionnaires from the patients we contacted (only 47%). Another limitation was that patients were asked to score the interval between sunlight exposure and occurrence as well as persistence of PLE lesions in a photosensitivity questionnaire (characteristics that were not determined/confirmed in a clinical photoprovocation assay) and also had to recall their disease course retrospectively. A third limitation was the introduction and use of better sun protection measures (including more effective broadband sunscreens with high UVA protection) during the period covered by our study and their possible contribution to the notion among some of our patients that PLE (most often caused by UVA wavebands) (10) had improved or even disappeared over time. Finally, the relatively low number of males in our study population limited the statistical power of our sex-based comparisons. Nonetheless, this study matches well in size and follow-up with the largest previous study so far on the course of PLE (39). However, in contrast to that previous study, in which some of the 94 patients developed associated diseases (including LE, actinic reticuloid PLE, or unusual forms of PLE such as prurigo-, solar urticaria-, or hydroa vaccinforme-like PLE) over time (39), our study had a more uniform PLE patient population.

In summary, this analysis revealed a long-term course of PLE. Though the disease improved in a substantial number of patients (i.e., 77% of females and 59% of males) over the years, it took 25 years until one third of patients had normalized from PLE. The persistence of skin lesions for more than 1 week under daily life conditions may predict a prolonged course of the disease over the years. However, the strength of lesion persistence as predictive factor needs to be assessed in further studies, possibly by combining data from different centers in a registry, like the Austrian Cooperative Registry for Photodermatoses. Such studies should also access the success or failure of photohardening and how this affects the long-term course of the disease. Moreover, how this all relates to the pathophysiology of the disease (for example, the failure of neutrophilic infiltration and other disturbances) (41) remains to be determined.

## Data Availability Statement

The questionnaires of the study and raw data supporting the conclusions of this article will be made available by the authors, without undue reservation.

## Ethics Statement

The studies involving human participants were reviewed and approved by Ethics Committee of the Medical University of Graz. The patients/participants provided their written informed consent to participate in this study.

## Author Contributions

AG-W: conceptualization-equal, data curation-equal, formal analysis-equal, investigation-equal, project administration-equal, validation-equal, writing-original draft-equal, and writing-review and editing-equal. TS: data curation, investigation-equal, project administration-supporting, and writing-review and editing-supporting. TG: data curation, formal analysis-equal, investigation, validation-equal, visualization-equal, and writing-review and editing-equal. FL: data curation, investigation, and writing-review and editing-equal. HR: data curation-supporting, investigation-supporting, and project administration-supporting. AH: data curation-equal, investigation-equal, and writing-review and editing-equal. FQ: formal analysis-equal, validation-equal, visualization-equal, writing-review and editing-equal. PW: conceptualization-lead, data curation-equal, formal analysis-lead, investigation-lead, methodology-lead, project administration-lead, resources-equal, supervision-lead, validation-lead, visualization-lead, writing-original draft-equal, and writing-review and editing-lead. All authors contributed to the article and approved the submitted version.

## Conflict of Interest

The authors declare that the research was conducted in the absence of any commercial or financial relationships that could be construed as a potential conflict of interest.
